# Flower power: Floral and resource manipulations reveal how and why reproductive trade‐offs occur for lowbush blueberry (*Vaccinium angustifolium*)

**DOI:** 10.1002/ece3.3109

**Published:** 2017-06-15

**Authors:** Alex W. Bajcz, Francis A. Drummond

**Affiliations:** ^1^ School of Biology and Ecology University of Maine at Orono Orono ME USA

**Keywords:** angiosperms, architectural constraints, carbon limitation, flower removal, nitrogen limitation, nonlinearity, reproductive ecology, theoretical ecology, trade‐offs

## Abstract

Plant reproductive trade‐offs are thought to be caused by resource limitations or other constraints, but more empirical support for these hypotheses would be welcome. Additionally, quantitative characterization of these trade‐offs, as well as consideration of whether they are linear, could yield additional insights. We expanded our flower removal research on lowbush blueberry (*Vaccinium angustifolium*) to explore the nature of and causes of its reproductive trade‐offs. We used fertilization, defoliation, positionally biased flower removal, and multiple flower removal levels to discern why reproductive trade‐offs occur in this taxon and to plot these trade‐offs along two continuous axes. We found evidence through defoliation that vegetative mass per stem may trade off with reproductive effort in lowbush blueberry because the two traits compete for limited carbon. Also, several traits including ripe fruit production per reproductive node and fruit titratable acidity may be “sink‐limited”—they decline with increasing reproductive effort because average reproductive structure quality declines. We found no evidence that reproductive trade‐offs were caused by nitrogen limitation. Use of reproductive nodes remaining per stem as a measure of reproductive effort indicated steeper trade‐offs than use of the proportion of nodes remaining. For five of six traits, we found evidence that the trade‐off could be concave down or up instead of strictly linear. *Synthesis*. To date, studies have aimed primarily at identifying plant reproductive trade‐offs. However, understanding how and why these trade‐offs occur represent the exciting and necessary next steps for this line of inquiry.

## 1 INTRODUCTION

In nature, two traits may negatively covary, with one increasing as the other decreases. This is deemed a “trade‐off” when either of two conditions is met. One is that the traits utilize the same limiting resource(s) such that high investment in both is infeasible. For example, production of pollen may nitrogen‐limit seed production and vice versa (Lehtilä & Ehrlén, 2005). Alternatively, the traits may contribute contrastingly to fitness such that one is more fitness‐generating in a given context. In perennials, sexual reproduction and vegetative expansion may trade off thusly, as heavy investment in only one at a time is typical (Sandvik, 2001). Either way, trade‐offs occur when one trait's increase is seen as *causing* the other's decrease. Given that resource limitation is common and that contexts will tend to favor one or another strategy, trade‐offs are likely ubiquitous (Hartemink, Jongejans, & de Kroon, 2004). Understanding trade‐offs, then, may reveal some of the ecological and evolutionary bases for observable plant phenotypes.

Reproduction lies at the center of one key set of trade‐offs in plants. For angiosperms, reproduction trades off with many other traits because, while it has high costs (Aragón, Méndez, & Escudero, 2009; Obeso, 2002), it is also central to fitness (Godschalx, Stady, Watzig, & Ballhorn, 2016). Recent efforts have identified many of the most common reproductive trade‐offs exhibited by angiosperms (see Bajcz, 2016; Chapter 1 for a summary), but we see value in pursuing three further lines of inquiry concerning these trade‐offs: (1) What causes these trade‐offs? (2) At what rates do other traits decline as reproductive effort increases? and (3) Are these rates constant along the range of a taxon's reproductive effort or do they vary? These questions are important because they may move us closer to a theory for why plants reproduce as they do (Houle, 2002).

### 1.1 Trade‐off causes—resource limitation

Reproductive trade‐offs are often thought to result from resource limitations, the logic being if plants had enough resources and could thus invest more into both traits involved, they would do so (Van Drunen & Dorken, 2012). However, this hypothesis has only been supported to a point for many known trade‐offs. For example, in a study with a different focus (Emms, 1996), *Toxicoscordion paniculatus* plants subjected to flower removal (a reduction in reproductive effort) produced more seeds than plants with natural reproductive effort levels, indicating that reproductive effort and seed production normally trade off in this taxon. Further, defoliated plants produced fewer seeds and nitrogen‐fertilized plants produced more seeds than control plants. These results suggest carbon and nitrogen availability, respectively, may normally limit seed production in this taxon.

It may be tempting to conclude that high reproductive effort *caused* seed production to be carbon‐ and/or nitrogen‐limited for *Toxicoscordion paniculatus*, but these results have not established such causality. The fertilization and flower removal treatments were not crossed, for example. While unlikely, it is conceivable that fertilization would have increased seed production equally in all cases—that is, seed production could be nitrogen‐limited *irrespective* of reproductive effort. Thus, to establish that high reproductive effort *causes* the resource limitation of another trait, it is necessary but insufficient to demonstrate that the two traits trade off and that these traits also covary with resource availability. It must additionally be shown that resource limitation of the other trait is *exacerbated* by increased reproductive effort—for example, that fertilization increases seed production *more* in plants with higher reproductive effort (which should be more nitrogen‐limited, if high reproductive effort leads to nitrogen scarcity) than in plants subjected to partial flower removal (which should thus be less nitrogen‐limited). This observed treatment interaction would provide stronger inference that reproductive effort causes the resource limitation that leads to the trade‐off in question.

### 1.2 Trade‐off causes—sink constraints

Reproductive trade‐offs have also been attributed to “temporal” (Brown & McNeil, 2006; Emms, 1996; Ishii & Sakai, 2002) and/or “architectural” (Guitián, Guitián, & Medrano, 2001; Pritchard & Edwards, 2005; Vallius, 2000) constraints. Briefly, these ideas propose that as more total sinks are produced, their average ability to effectively access or use available resources declines, either because earlier sinks inhibit later sinks (“temporal constraints”) or because some sinks are in a disadvantaged location (“architectural constraints;” Bajcz, 2016; see Chapter 1). We unify these ideas here under “sink limitation.” One form of sink limitation occurs when some sinks have poorer vasculature, impeding their access to resources even when these are otherwise available, which distinguishes this situation from resource limitation to some extent (Wesselingh & Arnold, 2003).

Because of their high fitness significance, reproductive sinks may be more advantaged than vegetative sinks on average (Godschalx et al., 2016; Tewari, Buonaccorsi, & Averill, 2014), although reproductive sinks may themselves vary in quality (Brown & McNeil, 2006). If some reproductive sinks are highly advantaged, as appears true for *Vaccinium macrocarpon* (Brown & McNeil, 2006), reducing reproductive effort may not only increase some vegetative traits by increasing relative resource access for vegetative sinks but also increase the average of some reproductive traits as well by eliminating many underperforming sinks. In that case, we would expect targeted removal of advantaged sinks, whichever these are, to have discernably contrasting effects to removal of disadvantaged sinks. So long as advantage and position covary, as may be true for *Vaccinium* spp. (Brown & McNeil, 2006), we hypothesized that targeted removal of sinks according to stem position should yield differences in advantage also.

### 1.3 Trade‐off rate and constancy

Our second two questions concern the rates at which reproductive trade‐offs occur and whether these rates are constant. Only a few studies we have found have addressed these specific questions (*e.g*., Sletvold & Ågren, 2015), likely because most previous flower removal studies have had motives differing from ours. In service of their questions, these studies have used binary (flower removal vs. nonremoval; *e.g*., Folke & Delph, 1997) or ordinal (proportions of flower removal; *e.g*., Godschalx et al., 2016) representations of their removal treatments in their analyses. However, to observe the rate at which the other trait involved declines as reproductive effort increases, as well as to determine whether this rate varies along the range of reproductive effort, we felt we needed to diverge from past work in two ways. First, we predicted use of a quantitatively continuous measure of reproductive effort would produce a better and more accurate fit to the data for each trade‐off. Second, we predicted a nonlinear regression approach could fit each trade‐off at least as well as a linear one because trade‐offs may often be nonlinear (Sletvold & Ågren, 2015). We also predicted our model fit would be improved by including a wide range of reproductive effort levels, which we could achieve by using a range of flower removal levels.

Here, we continued our flower removal research on lowbush blueberry (*Vaccinium angustifolium*; Bajcz & Drummond, in press) to answer the questions posed above. We focused on six traits we have already shown to trade off with reproductive effort for this taxon: vegetative mass per stem, surface area per leaf, ripe fruit produced per reproductive node (hereafter simply “node”), the proportion of successful nodes *(i.e*., those that produced >1 fruit by harvest), ripe fruit dry:fresh mass ratio, and fruit titratable acidity. We had three objectives: (1) Use four treatments (flower removal, positionally biased flower removal, defoliation, and fertilization) to determine the cause(s) of each trade‐off; (2) compare the fit of regressions using more ordinal (proportion of flowers remaining) versus more continuous (the number of flowers remaining) measures of reproductive effort for each trade‐off; and (3) compare the fits of linear versus exponential regressions for each trade‐off.

## 2 METHODS

### 2.1 Study system

Lowbush blueberry is a short‐statured, long‐lived perennial shrub managed commercially in Maine and Atlantic Canada for its fruit (Bell, Rowland, Stommel, & Drummond, 2010). Forests are converted to commercial fields via deforestation, leveling, and repeated burning or mowing until a carpet‐like mat of blueberry individuals (“clones”) have filled the space (Moore, 1994). These fields resemble other crop fields except they consist only of naturally recruited, wild genotypes (Rowland et al., 2012). Because of its unique characteristics, the lowbush blueberry agroecosystem may be ideal for studying reproductive trade‐offs in angiosperms for several reasons. First, because reproductive trade‐offs may be of interest to both ecologists (Sletvold & Ågren, 2015) and agronomists (Elle, 1996), the trade‐offs that occur within lowbush blueberry fields may be similar to those displayed by both conventionally managed and wild taxa. Further, while we could expect lowbush blueberry to reproduce relatively conservatively because it is long‐lived, woody, and perennial (Kaur, Percival, Hainstock, & Privé, 2012; Sletvold & Ågren, 2015), in managed fields, it can nevertheless produce tens of millions of flowers per hectare. Study of a conservative taxon displaying high reproductive effort may reveal trade‐offs and characteristics of these trade‐offs that may not be discernable through in situ studies of other wild taxa with lower reproductive effort levels.

### 2.2 Field and laboratory methodology

#### 2.2.1 Experimental design

Over 2 years (2014–15), clones (genets) of *V. angustifolium* were selected for study at the University of Maine's Blueberry Hill Farm (Bajcz & Drummond, in press). Because commercial lowbush blueberry fields are managed on a 2‐year cycle in which all aboveground biomass is destroyed after each harvest (Bell, Rowland, Smagula, & Drummond, 2009), it was not possible to use the same clones across years. As such, we selected 22 clones in 2014 and 31 new clones in 2015. The difference between years reflected our singular focus in 2014 and our split focus in 2015; our 2014 field work was devoted only to elucidation of the causes behind reproductive trade‐offs in lowbush blueberry (objective 1), whereas our 2015 field work sought to additionally characterize the rates and constancies associated with these trade‐offs (objectives 2 and 3).

**Figure 1:**
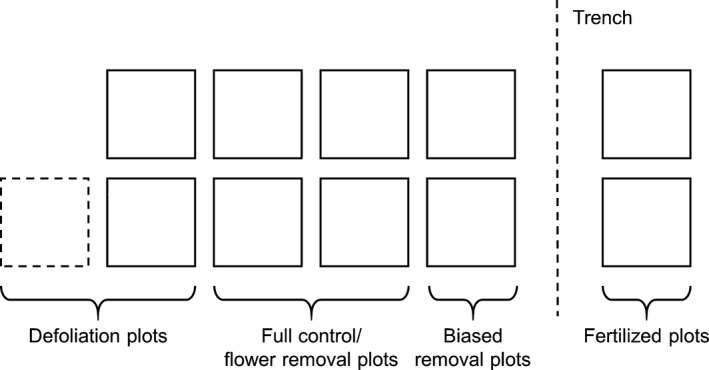
Treatment assignment schematic for study plots used to address objective 1 (*n* = 33 clones; see 1). Defoliated plots were subjected to partial or near‐total defoliation just following bloom. The partial defoliation + flower removal treatment was only performed in 2014 (dashed line plot). Biased removal plots were subjected to removal of reproductive nodes from the bottoms of stems upward or the tops of stems downward. Fertilized plots received five doses of the foliar nitrogen fertilizer Coron^®^ at a total rate of 1.91 ml m^−2^ per dose over a ~6‐week period prior to harvest. The dashed line indicates a trench that was dug and filled with aluminum flashing between fertilizer plots and biased removal plots. Plot assignment was random within the zones depicted except that full‐control plots were always diagonal from one another, as were flower removal‐only plots

In service of our singular goal for that year, eleven plots were established in all 22 clones in 2014 (Figure 1). In 2015, by contrast, we split the 31 selected clones into two groups: one of 11 clones for objective 1 and one of twenty clones for objectives 2 and 3. In the group of 11 clones, we established ten plots each, much as we had done in 2014 (although one treatment combination performed in 2014 was not repeated in 2015; Figure 1). In the set of twenty clones, we established just four plots. All plots were 0.125 m^2^ in area. Plot boundaries were marked with cord running along the soil surface and wrapped around stakes at each plot corner. Only those stems whose bases were within the cord boundary were treated as within a given plot. Plots were arranged in a rectangle within each clone with two plots per row and however many columns needed.

To address objective 1, treatment combinations were assigned to plots within each clone as follows. In 2014, plots were assigned to be: (1) full control (no resource manipulations or flower removal; two per clone); (2) reproductive node removal (hereafter “flower removal” or just “removal;” two per clone); (3) basipetal flower removal (hereafter “top‐down removal”); (4) acropetal flower removal (hereafter “bottom‐up removal”); (5) foliar nitrogen fertilization but no removal; (6) fertilization plus flower removal; (7) near‐total, midseason defoliation but no removal; (8) partial defoliation plus flower removal; and (9) near‐total defoliation plus flower removal (Figure 1). In 2015, plot assignment was identical for the group of 11 clones except that partial defoliation plus flower removal (treatment 8 above) was not performed due to logistic constraints. Plots receiving similar treatments were grouped together but otherwise arranged randomly (Figure 1). To address objectives 2 and 3, plots in the four‐plot clones from 2015 were assigned to either 0.0, 0.2, 0.4, or 0.6 reproductive node removal, with these proportions randomly assigned to plots within each clone. All plots were spaced 0.1 m apart from one another except for fertilized plots, which were placed 0.25 m away from the next‐nearest plots. Additionally, a 30‐cm‐deep trench was cut between the fertilized plots and the rest and filled with aluminum flashing to prevent cross‐contamination of other plots with fertilizer. We cut this trench as far from our plots as possible and, although some stress was visible on stems very close to the trench, we did not see any evidence, visual or otherwise, that the trench had a negative impact on the health of our study plots.

#### 2.2.2 Treatment application and plot management

Prior to bloom, all reproductive nodes on all stems (ramets) in all plots were hand‐counted. In all removal plots, we then pinched off the appropriate proportion (0.2, 0.4, 0.6, or 0.7) of the reproductive nodes from every stem. The number of nodes removed from each stem was rounded to the nearest whole node with three exceptions: (1) stems with a single node were skipped in all cases; (2) in 0.7 removal plots, two‐node stems had only one node removed; and (3) removal was always rounded down for stems with a node number divisible by five. In the positionally biased removal plots, removal proceeded from the tops of stems downward or from the bottoms of stems upward, as appropriate. For stems with side branches, node position was judged based on absolute distance to the root collar. In all other removal plots, nodes were removed haphazardly with respect to position.

Fertilized plots received five doses of Coron^®^ (Helena Chemical Company, Collierville, TN) foliar nitrogen fertilizer (~28% N by volume), each dose seven to 10 days apart, from mid‐June through late July (Smagula & Kreider, 2009). Delivery rate was 1.91 ml Coron/m^2^ per dose (Smagula & Kreider, 2009), delivered with a handheld spray bottle. In defoliation plots, we pinched off all but the top three (“partial”) or one (“near‐total”) vegetative node(s) from each stem just following bloom (mid‐June). All plots received targeted herbicide, fungicide, and insecticide treatments only as needed to minimize the possibility of pest outbreaks (Yarborough, 2008). Honey bees were stocked during bloom at a rate of four hives/ha to ensure adequate pollination (Drummond, 2002). Otherwise, all plots were not manipulated further.

#### 2.2.3 Trait quantification

We collected a subsample of stems (8–10 stems per collection, or about 15%–20% of stems per plot, on average) from every plot at two time points during the growing season—bloom (late May/early June) and midseason (late June/early July). Then, at harvest (early August), all remaining stems were collected. Reproductive and vegetative tissues from each plot were separated and frozen at −20°C for trait quantification in the laboratory. The number of living reproductive nodes remaining on each stem was recorded prior to tissue separation.

In the laboratory, we quantified average vegetative mass per stem (g/stem) at midseason and at harvest by dividing total vegetative mass (*i.e.,* leaves plus new green stems) by the number of stems collected at each time point. We measured average surface area per leaf (cm^2^) by averaging surface area measurements from three healthy leaves chosen from each plot for both the midseason and harvest collection points. Area measurements were taken with a scanning image analyzer in 2014 (Agvision Monochrome System^®^; Decagon Devices Inc., Pullman Wash.) and a leaf area meter in 2015 (LI‐COR LI‐3000A Portable Leaf Area Meter^®^, LI‐COR, Inc., Lincoln, Nebr.). We had to switch devices because the former became unavailable. However, the data produced by the two devices were comparable.

Reproductive node success rate was calculated by dividing the number of nodes recovered from each plot across all collections by the initial number of nodes in each plot at the outset of the study following any removal carried out. For this trait, we coded nodes from the bloom and midseason collections as “successful” although some fraction of these may not have ultimately produced fruit, so we have likely overestimated the true success rate. We categorized all fruit at harvest as ripe (*i.e.,* with an entirely blue surface with no red, white, or green portions) or unripe, and the number of ripe fruit produced per node was derived by dividing the number of ripe fruit by the number of nodes remaining at harvest. We determined ripe fruit dry:fresh mass ratio by dividing the fresh mass (g) of four ripe fruit from each plot into their mass after being dried at 70°C for 72 hr. Lastly, in 2015 only, we estimated ripe fruit titratable acidity in percent citric acid equivalents by volume for each plot via titration of 1 ml fruit juice samples with a 0.005N sodium hydroxide solution to a pH endpoint of 8.2 (Bajcz & Drummond, in press).

### 2.3 Statistical analyses

All data were analyzed in R (R Core Team, 2017) with figures made using *ggplot2* (Wickham, 2009).

#### 2.3.1 Objective 1

To assess whether nitrogen, carbon, or sink limitation explained why each reproductive trade‐off was occurring, we performed a set of linear mixed‐effect regressions (package: *lme4*; function: *lmer*; Bates, Mächler, Bolker, & Walker, 2015), one for each trait. For this set of analyses only, we used data from the harvest collection instead of from the midseason collection for vegetative mass per stem and area per leaf to allow the full impacts of the defoliation and fertilization treatments, if any, to manifest. We also used data only from those clones designated for objective 1 (22 clones from 2014 and 11 clones from 2015; *N* = 352 plots) to reduce the number of factors needed in each model. We included clone and year as grouping factors in each model except year for the titratable acidity model because this trait was measured only in 2015.

Each model included three fixed factors used to assess the validity of the nitrogen, sink, and carbon limitation hypotheses, respectively: the removal‐by‐fertilization interaction factor, the direction of biased removal factor (coded numerically as −1 and 1 for bottom‐up removal and top‐down removal, respectively, and 0 for all other treatments), and the removal‐by‐defoliation interaction factor. We hypothesized that one or more of these factors would be significant for each trait. Main‐effect factors for flower removal (1 for removal and 0 for nonremoval), fertilization (1 for fertilization and 0 for nonfertilization), and defoliation were also included as required when including higher‐order factors, but these were not of primary interest. We determined that our partial defoliation treatment removed 0.67 as much vegetative mass, on average, as our near‐total defoliation treatment did. To represent this treatment as accurately as possible in the model, we coded defoliation numerically as 1, 0.67, and 0 for near‐total defoliation, partial defoliation, and no defoliation, respectively.

We also initially included two other covariates in each model. To account for variability in the removal treatment due to node rounding, we included “EST.REM,” which was the proportion of nodes that would have been removed from each plot if it had been a 0.7 removal plot. In practice, this covariate increased as the average number of nodes per stem in a plot increased, so it is also a measure of initial fecundity. A second covariate (“Biased Rem,” coded as 1 for both directions of biased removal and 0 for all other treatments) was included in the event that biased flower removal *itself*, irrespective of its direction (top‐down or bottom‐up) had a relationship with trait values. These two covariates were removed from each model in order of largest *p* value until any that remained were statistically significant (*i.e*., *p *<* *.05).

Each model was checked to ensure it met linear model assumptions. To better meet these assumptions, we natural log‐transformed vegetative mass per stem, area per leaf, and proportion node success prior to final analysis. One influential observation, an outlier (*i.e*., more than three standard deviations from the mean) for EST.REM, was removed from the models for ripe fruit per node, proportion node success, and fruit dry:fresh mass because it unduly influenced the significance of the EST.REM covariate. Additionally, an outlier for fruit dry:fresh mass was removed from the corresponding model because the (non)significance of several factors depended solely on its inclusion. We used the Kenward–Roger approximation for denominator degrees of freedom for these regressions (package: *pbkrtest*; Halekoh & Højsgaard, 2014). Fixed factors were treated as significant at *p *<* *.05. A conditional *R* ^2^ value for each model was obtained using the *sem.model.fits* function (package: *piecewiseSEM;* Lefcheck, 2015).

#### 2.3.2 Objective 2

For our second set of analyses to determine whether a more ordinal or a more continuous measure of reproductive effort would produce a better fit to the trade‐off data, we performed two linear mixed‐effect regressions for each trait (Bates et al., 2015). One model had the proportion of reproductive nodes remaining per plot after any node removal as a measure of reproductive effort, whereas the other had the average number of nodes remaining per stem as the reproductive effort measure instead. We predicted that the latter would produce a superior fit for each trade‐off. Here and in our third set of analyses, we included only data from 2015, both from the group of 20 clones subjected to multiple removal levels (20 clones, 80 plots) and from the full‐control and 0.7 removal‐only plots (11 clones, 44 plots) from all other clones from that year. We included clone as a grouping factor in these models and used the Kenward–Roger approximation to determine *p* values (Halekoh & Højsgaard, 2014); these were deemed significant at *p *<* *.05. To facilitate direct model comparisons, we report the standardized linear fixed‐effect regression coefficient (β_std._) for each model.

#### 2.3.3 Objective 3

In the third set of analyses to determine whether the reproductive trade‐offs under study could be nonlinear, we fit a pair of regressions for each trade‐off using the nonlinear regression function *nlsLM* in the package *minpack.lm* (Elzhov, Mullen, Spiess, & Bolker, 2015). In each pair of regressions, the average number of nodes remaining per stem was the sole fixed factor. Otherwise, the models took contrasting forms:

(1) $$Y=\beta_{0}+\beta_{1}X$$

(2) $$Y=\beta_{0}+\beta_{1}e^{\beta 2X}$$

In Equations (1) and (2), *Y* represents the data for the other trait involved in the trade‐off, *X* represents the reproductive effort data, and β_0_, β_1_, and β_2_ are regression coefficients. Equation (1) is the traditional linear function, whereas Equation (2) is an exponential‐family function. We chose this particular form for Equation (2) because it can fit both forms of concavity that trade‐offs are thought to exhibit—concave up and concave down (Sletvold & Ågren, 2015). When β_1_ **<* *0 and β_2_ > 0, the curve will be concave down, and when β_1_ > 0 and β_2_ **<* *0, the curve will be concave up. Additionally, in contrast to the quadratic‐family functions (*e.g*., *Y* = β_0_ + β_1_X + β_2_X^2^) used in the past to model trade‐off nonlinearity (*e.g*., Maust, Williamson, & Darnell, 1999, 2000; Sletvold & Ågren, 2015), this exponential function is assured to be monotonic (*i.e*., continuously decreasing) as reproductive effort increases.

We discovered, in initial diagnoses, that within‐clone data were highly auto‐correlated. Because the *nlsLM* function does not allow for inclusion of grouping factors, we first performed a linear mixed‐effect regression for each trait with no fixed factors and clone as a grouping factor (Bates et al., 2015). We extracted the residuals from these models and used them as our dependent trait data in the models described above. This sequential modeling approach satisfactorily eliminated the auto‐correlative effect. We derived 95% confidence intervals (CIs) for each model's regression coefficients using the bootstrapping function *nlsBoot* in the package *nlstools* (*n* = 999 iterations; Baty et al., 2015). The *nlsLM* function (Elzhov et al., 2015) frequently did not converge during bootstrapping for the exponential models, so we modified it to permit up to 1000 iterations per bootstrap, which allowed it to converge 100% of the time. This increased our conservativeness by allowing data distributions very unlike our real data to converge, widening the average width of the CIs. We deemed coefficients significant if 0 was not within their CI. We compared the fit of the linear and exponential models via Akaike's information criterion values corrected for small sample sizes (AICc) retrieved with the function *AICc* (package: *AICcmodavg*; Mazerolle, 2016). By convention, when AICc values differed by >2 points, we deemed the model with the lower value a superior fit (Burnham & Anderson, 2002). We tested for influence using a jackknife analysis performed via the *nlsJack* function (package: *nlstools*; Baty et al., 2015). Observations with high influence scores were double‐checked for accuracy and outlier status; we did not deem any observations inaccurate or unduly influential.

## 3 RESULTS

### 3.1 Objective 1: are reproductive trade‐offs caused by resource and/or sink limitation(s)?

#### 3.1.1 Vegetative traits

**Figure 2:**
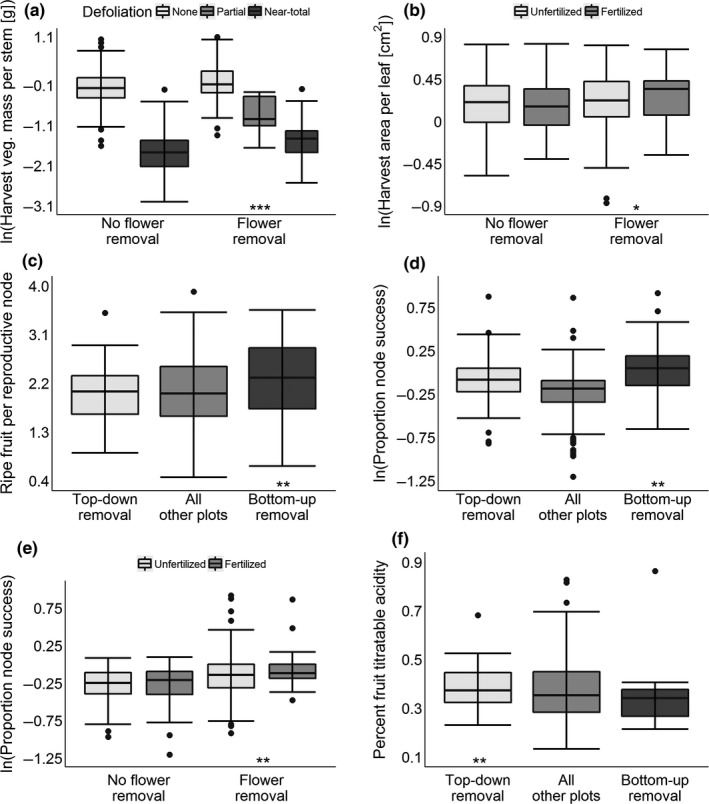
Results from a multi‐treatment study performed on plots of *Vaccinium angustifolium*. Treatments included removal of 0.7 of the reproductive nodes from each stem (all panels), midseason defoliation (panel a), foliar nitrogen fertilization (panels b and e), and biased node removal (*i.e*., removal of nodes from the bottoms of stems upward or the tops of stems downward; panels c, d, and f). Proportion node success was the number of reproductive nodes recovered from each plot divided by the postremoval number of nodes remaining. Significant interactions between defoliation or fertilization and flower removal are marked by groups of asterisks below the removal treatment group for which the defoliation/fertilization group mean was higher. Significant effects of removal direction are marked by groups of asterisks below the biased removal group with the higher mean. Significance is coded as follows: *0.1 ≥ *p *≥* *.05 (marginal significance); **0.5 ≥ *p *≥* *.001; ***p <* *.001. All *p* values are from mixed‐effect regressions with the Kenward–Roger approximation (Halekoh & Højsgaard, 2014). Whiskers represent a distance of 1.5 ×  IQR from the median

Not surprisingly, vegetative mass per stem at harvest was significantly reduced by defoliation (Table 1); near‐totally defoliated plots had just 19.8% the vegetative mass of intact plots on average (Figure 2a). Flower removal led to significantly more vegetative mass per stem as well—removal plots had 13.8% more vegetative mass, on average, than nonremoval plots overall (Table 1). Consistent with high reproductive effort causing carbon limitation of vegetative mass, we saw a significant interaction between defoliation and flower removal such that removal plots had 26.6% greater vegetative mass when defoliated than comparably defoliated nonremoval plots. Of the remaining fixed factors in the model for this trait, only the EST.REM covariate was significant, so we found no evidence that this trait was nitrogen‐ or sink‐limited by high reproductive effort.

**Table 1:** Linear mixed‐effect regression results relating *Vaccinium angustifolium* trait values to several treatments: reproductive node removal, positionally biased node removal, nitrogen fertilization, and midseason defoliation

Fixed effects	ln(Harvest veg. mass per stem (g))			ln(Harvest area per leaf (cm^2^))			Ripe fruit per reproductive node		
	β	*t* a	*p* b	β	*t*	*p*	β	*t*	*p*
Intercept	−1.039	−5.153	**<.001**	−0.281	−1.577	.187	2.050	8.199	.068
Removal	0.129	2.897	**.004**	0.004	0.134	.894	0.076	1.222	.223
Fertilization	−0.112	−1.750	.081	−0.060	−1.354	.177	0.013	0.144	.886
Defoliation	−1.621	−21.721	**<.001**	−0.037	−0.834	.405	−0.667	−7.548	**<.001**
Rem. × Fert.	0.062	0.730	.466	0.107	1.782	.076	0.036	0.301	.763
Rem. × Defol.	0.236	2.861	**.005**	0.078	1.348	.179	0.133	1.150	.251
Biased Rem. Dir.^c^	0.042	1.147	.252	−0.009	−0.342	.733	0.158	3.042	**.003**
*Biased Rem*.^d^	–	–	–	–	–	–	–	–	–
*EST.REM* e	1.539	4.772	**<.001**	0.763	3.550	**<.001**	–	–	–
*Cond. R* ^*2*^ f	0.857			0.582			0.679		
*N*	351			351			350		

Fixed effects	ln(Proportion node success)^g^			Ripe fruit dry:fresh mass			Percent fruit titratable acidity^h^		
	β	*t*	*p*	β	*t*	*p*	β	*t*	*p*
Intercept	−0.270	−3.473	.146	0.166	13.932	**<.001**	0.397	6.137	**<.001**
Removal	0.063	1.742	.083	0.006	3.340	**.001**	0.059	3.916	**<.001**
Fertilization	−0.038	−0.855	.393	−0.006	−2.158	**.032**	0.042	2.075	**.041**
Defoliation	−0.064	−1.439	.151	−0.012	−4.337	**<.001**	0.068	3.351	**.001**
Rem. × Fert.	0.148	2.360	**.019**	0.003	0.874	.338	−0.047	−1.633	.106
Rem. × Defol.	−0.033	−0.530	.597	−0.005	−1.502	.134	−0.018	−0.589	.558
Biased Rem. Dir.	0.066	2.546	**.011**	−0.001	−0.782	.435	−0.032	−2.507	**.014**
*Biased Rem*.	0.166	4.568	**<.001**	–	–	–	–	–	–
*EST.REM*	–	–	–	−0.063	−4.451	**<.001**	–	–	–
*Cond. R* ^*2*^	0.475			0.623			0.937		
*N*	351			350			97		

a *t* statistics were estimated using the Kenward–Roger approximation (Halekoh & Højsgaard, 2014).

b Probability values were treated as significant at *p *<* *.05 (in bold).

c The direction of biased removal was coded −1 and 1 for top‐down‐removal and bottom‐up‐removal plots, respectively, and 0 for all other plot types.

d The biased removal term was coded 1 for both biased removal plots and 0 for all other plot types (only included when significant).

e EST.REM was included as a covariate only when significant to account for variation in reproductive node‐removal treatment intensity between plots (see text).

f The conditional *R* ^2^ was obtained using the function *sem.model.fits* (Lefcheck, 2015).

g The proportion node success was the number of reproductive nodes recovered from a plot divided by the number of nodes remaining following the initial node‐removal treatment.

h Percent titratable acidity is in citric acid equivalents.

Average surface area per leaf at harvest correlated significantly with our EST.REM covariate (Table 1). We also observed evidence of an interaction between removal and fertilization for this trait, but with removal plots having 11.3% higher, not lower, leaf area values when fertilized than fertilized nonremoval plots (Figure 2b). This is the opposite pattern expected if high reproductive effort was causing leaf area to be nitrogen‐limited. We also observed no evidence that area per leaf was carbon‐ or sink‐limited.

#### 3.1.2 Reproductive traits

Near‐totally defoliated plots produced 0.67 fewer ripe fruit per node by harvest than intact plots, on average (Table 1). Also, bottom‐up removal plots produced 0.32 more ripe fruit per node than top‐down removal plots on average (Figure 2c), suggesting this trait may be sink‐limited under high reproductive effort due to poorer average performance of more basal reproductive nodes. No other factors were significant for this trait, indicating it may not be carbon‐ or nitrogen‐limited by high reproductive effort.

Biased removal plots, when considered in tandem, had 18.1% higher node success rates than all other plot types (Table 1). Beyond this, bottom‐up removal plots had 14.1% higher rates than top‐down plots (Figure 2d), indicating this trait may be sink‐limited when reproductive effort is high, again due to poorer relative performance of more basal nodes. We also observed a significant removal‐by‐fertilization interaction for this trait, but, again, fertilized removal plots had 16.0% higher, not lower, success rates than comparable nonremoval plots (Figure 2e). As such, this trait did not show evidence of nitrogen limitation, nor did we find evidence that it was carbon‐limited because no other fixed factors were significant.

#### 3.1.3 Fruit traits

Ripe fruit dry:fresh mass correlated negatively with our EST.REM covariate (Table 1). Beyond that, removal plots had significantly higher dry:fresh mass values than nonremoval plots by 0.006, on average (Table 1). Fertilization decreased fruit dry:fresh mass by roughly the same amount, whereas near‐total defoliation decreased it by roughly double that amount (Table 1). No other factors were significant for this trait, indicating it is not apparently sink‐, carbon‐, or nitrogen‐limited by high reproductive effort.

Ripe fruit titratable acidity (in percent citric acid equivalents by volume) was increased by flower removal, fertilization, and defoliation (Table 1). We also observed a significant relationship between titratable acidity and biased removal direction, with top‐down removal plots having 0.064% higher titratable acidity values than bottom‐up removal plots on average (Figure 2f). This supports the hypothesis that this trait is sink‐limited when reproductive effort is high due to lower average acidity in fruits produced by more apical nodes. No other fixed factors were significant in this regression, although fertilized nonremoval plots had a nonsignificant tendency toward higher average titratable acidity values than fertilized removal plots (*p *=* *.106), as might be expected under nitrogen limitation.

### 3.2 Objective 2: which measure of reproductive effort best fits each trade‐off?

**Figure 3:**
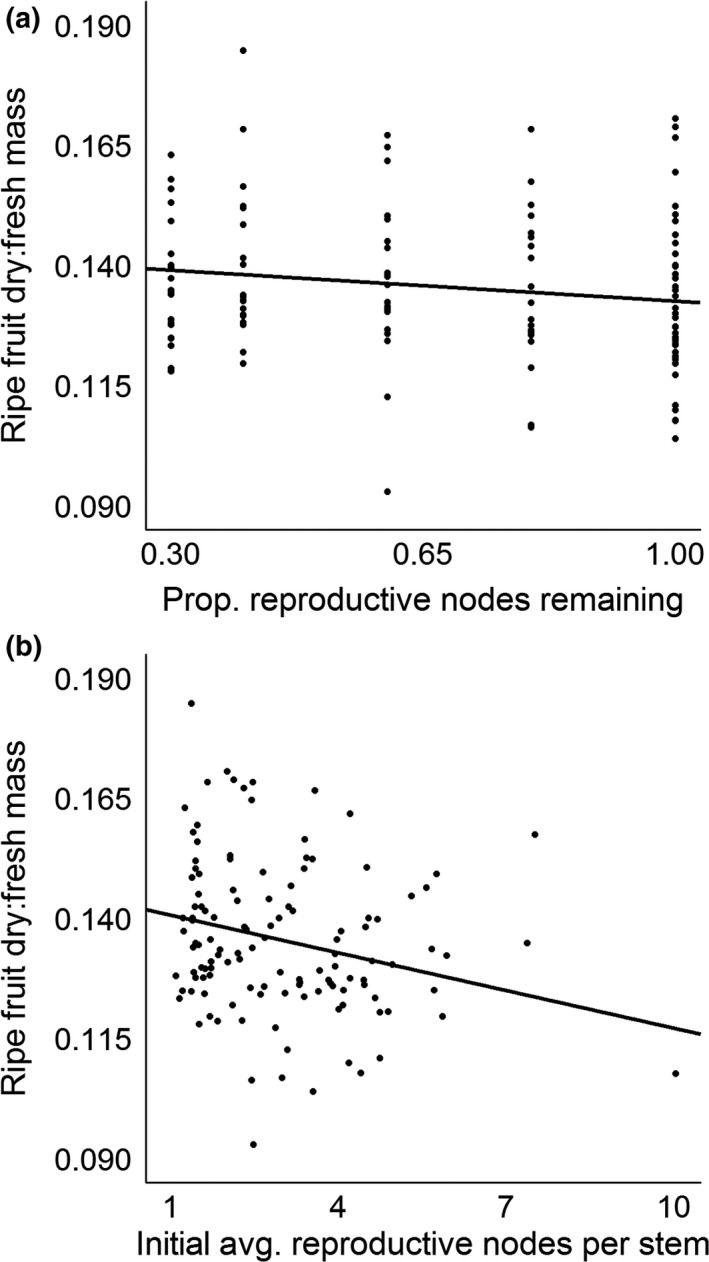
Relationships between average *Vaccinium angustifolium* ripe fruit dry:fresh mass and the proportion (panel a) and number (panel b) of reproductive nodes remaining following a node‐removal treatment at the onset of the study. Both relationships were statistically significant (both *p* values < .05), but the slope estimate for the latter (β = −0.242) was more negative than for the former (β = −0.147). The latter is still significant (*p *=* *.011) with the rightmost observation removed

In our second set of analyses, our more ordinal measure of reproductive effort, the proportion of reproductive nodes remaining after our removal treatments, was negatively correlated with four of our six traits (Table 2): vegetative mass per stem; ripe fruit produced per node; fruit dry:fresh mass; and fruit titratable acidity. By comparison, the average postremoval number of nodes remaining per stem, our more continuous measure of reproductive effort, was negatively correlated with five traits: the four listed above as well as proportion node success. An exemplary visual comparison of the two competing models is provided in Figure 3 for fruit dry:fresh mass. Neither measure correlated significantly with area per leaf. Importantly, the standardized regression coefficients (β_std._) from the models using the more continuous measure of reproductive effort were more negative than those from the competing regressions for all six traits, indicating a more negative trade‐off in each case.

**Table 2:** Linear mixed‐effect regression results relating six other *Vaccinium angustifolium* traits to two measures of reproductive effort: the proportion of reproductive nodes remaining following a flower removal treatment or the average number of nodes remaining per stem. Data are from plots subjected to levels of node removal ranging from 0 to 0.7

Predictor	Midseason veg. mass per stem (g)			Midseason area per leaf (cm^2^)			Ripe fruit per reproductive node		
	β^a^	*t*	*p* b	β	*t*	*p*	β	*t*	*p*
Prop. nodes remaining	−0.177	−3.381	**.001**	0.011	0.161	.872	−0.074	−2.349	**.021**
Nodes remaining per stem	−0.205	−3.099	**.003**	−0.048	−0.572	.569	−0.105	−2.560	**.012**

Predictor	Proportion node success^c^			Ripe fruit dry:fresh mass			Percent fruit titratable acidity^d^		
	β	*t*	*p*	β	*t*	*p*	β	*t*	*p*
Prop. nodes remaining	−0.096	−1.725	.088	−0.147	−2.305	**.023**	−0.093	−2.922	**.004**
Nodes remaining per stem	−0.144	−2.092	**.039**	−0.242	−3.186	**.002**	−0.139	−3.402	**.001**

a Regression coefficients have been mean‐standardized.

b Probability values were estimated using the Kenward–Roger approximation (Halekoh & Højsgaard, 2014) and were treated as statistically significant at *p *<* *.05 (in bold).

c The proportion node success was the number of reproductive nodes recovered from a plot divided by the number of nodes remaining just following the node‐removal treatment.

d Percent titratable acidity is in citric acid equivalents.

### 3.3 Objective 3: are reproductive trade‐offs linear or nonlinear?

#### 3.3.1 Vegetative traits

**Figure 4:**
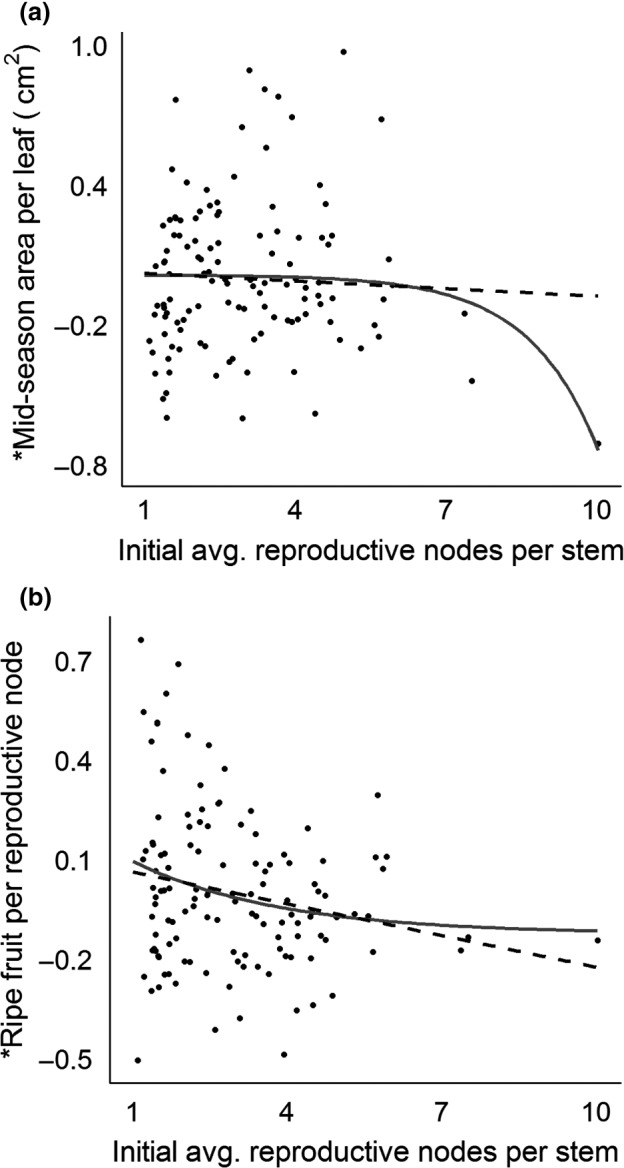
Linear (dashed lines) and exponential (solid curves) relationships between two exemplary *Vaccinium angustifolium* traits (area per leaf in panel a and ripe fruit per node in panel b) and the average number of reproductive nodes remaining per stem after a node‐removal treatment at the onset of the study. *Y*‐axis values on both panels are residuals from linear mixed‐effect regressions used to remove the confounding influence of including multiple plots from within the same genotype. All models upon which the lines/curves are based on had statistically significant regression coefficients at α = 0.05 except the line in panel a

In our last set of analyses, all regression coefficients in both the linear and exponential models were significant for the vegetative‐mass‐per‐stem‐versus‐reproductive‐effort trade‐off (Table 3). The models had AICc values within 2, indicating comparable fit. The exponential model coefficients indicated the trade‐off could be concave down (*i.e*., β_1_ **<* *0 and β_2_ > 0). For the midseason‐area‐per‐leaf‐versus‐reproductive‐effort trade‐off, the linear model was a poor fit to the data and had no significant coefficients. Meanwhile, the exponential model was a good fit, with all but the asymptote coefficient (β_0_) differing significantly from 0. This comparison is shown visually as an exemplar in Figure 4a. The exponential model's AICc value was more than 2 units lower, indicating a significantly better fit, and that model indicated this trade‐off was concave down.

**Table 3:** Exponential and linear regression results relating six other *Vaccinium angustifolium* traits to reproductive effort, as measured via the number of reproductive nodes remaining per stem just after a flower removal treatment

	Midseason veg. mass per stem (g)			Midseason area per leaf (cm^2^)			Ripe fruit per reproductive node		
	Estimate^a^	2.5%^b^	97.5%	Estimate	2.5%	97.5%	Estimate	2.5%	97.5%
Exp.^c^									
β_0_	**0.155 × 10** ^−**1**^	1.10 × 10^−3^	9.53 × 10^1^	1.47 × 10^−2^	−4.98 × 10^−2^	2.36 × 10^1^	−**1.25 × 10** ^−**1**^	−8.80 × 10^1^	−.66 × 10^−3^
β_1_	−**7.00 × 10** ^−**2**^	−9.52 × 10^1^	−1.98 × 10^−9^	−**5.11 × 10** ^−**4**^	−2.35 × 10^1^	−4.42 × 10^−13^	**3.12 × 10** ^−**1**^	1.35 × 10^−1^	9.87 × 10^1^
β_2_	**0.237 × 10** ^−**1**^	4.10 × 10^−4^	1.90 × 10^0^	**7.27 × 10** ^−**1**^	6.70 × 10^−4^	2.71 × 10^0^	−**3.48 × 10** ^−**1**^	−3.59 × 10^0^	−2.64 × 10^−4^
AICc^d^	46.60			*78.79*			−4.17		
Linear									
β_0_	**1.48 × 10** ^−**1**^	3.63 × 10^−1^	2.60 × 10^−1^	3.26 × 10^−2^	−1.03 × 10^−1^	1.63 × 10^−1^	**9.45 × 10** ^−**2**^	9.96 × 10^−3^	1.76 × 10^−1^
β_1_	−**4.91 × 10** ^−**2**^	−8.27 × 10^−2^	−1.63 × 10^−2^	−1.09 × 10^−2^	−4.75 × 10^−2^	2.78 × 10^−2^	−**3.18 × 10** ^−**2**^	−5.75 × 10^−2^	−6.07 × 10^−3^
AICc	45.36			81.74			−5.55		
*N*	120			121			124		

	Proportion node success^e^			Ripe fruit dry:fresh mass			% fruit titratable acidity^f^		
	Estimate	2.5%	97.5%	Estimate	2.5%	97.5%	Estimate	2.5%	97.5%
Exp.									
β_0_	−**2.45 × 10** ^−**1**^	−6.63 × 10^1^	−4.59 × 10^−3^	−**1.19 × 10** ^−**2**^	−5.39 × 10^0^	−1.08 × 10^−3^	**3.3 × 10** ^−**2**^	1.8 × 10^−3^	2.3 × 10^0^
β_1_	**2.83 × 10** ^−**1**^	4.73 × 10^−2^	7.14 × 10^1^	**2.01 × 10** ^−**2**^	1.11 × 10^−2^	5.75 × 10^0^	−**1.2 × 10** ^−**2**^	−2.3 × 10^0^	−7.6 × 10^−4^
β_2_	−**4.99 × 10** ^−**1**^	−3.87 × 10^0^	−2.73 × 10^−5^	−**1.90 × 10** ^−**1**^	−2.75 × 10^0^	−2.88 × 10^−4^	**3.1 × 10** ^−**1**^	4.1 × 10^−3^	1.0 × 10^0^
AICc	−204.04			−757.61			−338.03		
Linear									
β_0_	3.45 × 10^−2^	−2.48 × 10^−3^	7.72 × 10^−2^	**5.70 × 10** ^−**3**^	1.80 × 10^−3^	9.83 × 10^−3^	**3.4 × 10** ^−**2**^	1.2 × 10^−2^	5.7 × 10^−2^
β_1_	−1.16 × 10^−2^	−2.35 × 10^−2^	7.06 × 10^−4^	−**1.92 × 10** ^−**3**^	−3.15 × 10^−3^	−7.17 × 10^−4^	−**1.2 × 10** ^−**2**^	−1.9 × 10^−2^	−4.7 × 10^−3^
AICc	−*206.15*			−759.58			−339.76		
*N*	124			124			114		

Regressions were performed on the residuals of a linear mixed‐effect regression (function *lmer*; Bates et al., 2015; see 1.1).

a A parameter estimate was deemed significant (in bold) if 0 fell outside its corresponding 95% confidence interval.

b 95% confidence intervals were determined using a nonparametric bootstrap using a modified version of the function *nlsBoot* (Baty et al., 2015).

c The exponential model was of the form Y = β_0_ + β_1_e^β2X^. The linear model was of the form Y = β_0_ + β_1_X.

d The Akaike's information criterion corrected for small sample sizes (AICc) was determined using the function *AICc* (Mazerolle, 2016). When AICc values differed by more than 2 for competing models, the model with the lower value was considered the better fit (in italics; Burnham & Anderson, 2002).

e The proportion node success was the number of reproductive nodes recovered from a plot divided by the number of nodes remaining just following the node‐removal treatment.

f Percent titratable acidity is in citric acid equivalents.

Inspection of Figure 4a suggests that a single data point (at ~10 nodes/stem) may exert influence on the fit of the exponential model. A post hoc jackknife analysis did show that removal of this data point would change the regression coefficient estimates considerably. However, it can also be observed that the three data points furthest to the right (7 + nodes per stem) all suggest a decline in midseason area per leaf at high levels of reproductive effort. Further, our jackknife analysis indicated that the furthest‐right data point ranks only fifth in overall influence on the regression coefficient estimates and that more than 50 of the 121 observations had a noteworthy level of influence over the coefficient estimates of this model. Thus, we have no reason to believe this data point, or any other single observation, is inaccurate, abnormal, or unreflective of reality. Still, we caution our results should be treated tentatively.

#### 3.3.2 Reproductive traits

All coefficients in both models were significant for the ripe‐fruit‐production‐per‐node‐versus‐reproductive‐effort trade‐off, and the AICc values for the models were within two units (Table 3), indicating comparable fit. The exponential model indicated this trade‐off may be concave up (Figure 4b). For proportion node success, neither of the linear model's coefficients were significant, whereas all of the exponential model's coefficients differed significantly from 0. For this trade‐off, the model indicated a potential concave‐up shape. However, the linear model's AICc value was more than 2 units less than that of the exponential model, indicating the former was a better fit (Table 3), which suggests neither model may be adequate for this trade‐off.

#### 3.3.3 Fruit traits

Both models fit the data well for the fruit‐dry:fresh‐mass‐versus‐reproductive‐effort and the titratable‐acidity‐versus‐reproductive‐effort trade‐offs, with all coefficients in all models significantly differing from 0 (Table 3). For fruit dry:fresh mass, the exponential model indicated a possible concave‐up shape, although the linear model was deemed a comparable fit to the data based on AICc values (Table 3). The results were similar for titratable acidity except that the exponential model indicated a possible concave‐down shape instead (Table 3).

## 4 DISCUSSION

### 4.1 Objective 1: causes of reproductive trade‐offs in lowbush blueberry

We used flower removal of multiple types coupled with nitrogen fertilization and midseason defoliation to test hypotheses for why reproductive trade‐offs occur for lowbush blueberry. In keeping with our past results (Bajcz & Drummond, [Link]), flower removal, alone or in combination with other treatments, led to increases in five of six traits under study. The sixth trait, ripe fruit production per node, did not increase in response to standard flower removal in this study but did increase in response to biased flower removal (Table 1). Increases in vegetative mass (Hartemink et al., 2004; Maust et al., 1999), leaf area (Karlsson, Andersson, & Svensson, 2006; Maust et al., 1999, 2000), fruit ripening rate (Maust et al., 1999, 2000; Valantin‐Morison, Vaissière, Gary, & Robin, 2006), and fruit dry mass (Vallius, 2000) have all been reported in previous flower removal studies. However, we have found no studies besides ours that have reported significant removal effects on proportion node success or fruit titratable acidity.

Importantly, we found little evidence that reproductive effort trades off with other lowbush blueberry traits because of nitrogen limitation. First, while fertilization did lead to higher fruit titratable acidity across all plots, indicating nitrogen availability may somehow limit this trait overall, fertilization instead seemed to decrease fruit dry:fresh mass and vegetative mass per stem (*p *=* *.081). Beyond that, we found no interactions between flower removal and fertilization in the expected direction for any trait such that fertilization increased trait values more for nonremoval plots than for removal plots. In fact, removal plots actually had higher proportional node success and a trend toward (*p *=* *.076) higher area‐per‐leaf values when fertilized than fertilized nonremoval plots, the reverse of the pattern we expected to observe.

Our findings are also not consistent with a hypothesis that reproductive trade‐offs in lowbush blueberry occur due to carbon limitation. We did observe, though, that removal plots had greater vegetative mass per stem at harvest when defoliated than comparable nonremoval plots, indicating carbon limitation may constrain vegetative effort in lowbush blueberry as a direct result of high reproductive effort. Defoliation decreased ripe fruit production per node and fruit dry:fresh mass, indicating carbon availability could limit these traits in lowbush blueberry overall, but defoliation actually increased titratable acidity, which would not support a carbon limitation hypothesis for this trait. Most crucially, for no other trait besides vegetative mass per stem were nonremoval plots impacted more by defoliation than removal plots, as we would have expected. As such, while defoliation may cause (or worsen) carbon limitation in lowbush blueberry for some traits, it may do so largely irrespective of reproductive effort.

In contrast to the results above, we found evidence that three lowbush blueberry traits of the six studied may be sink‐limited by high reproductive effort. Plots with top nodes remaining had higher rates of node success and ripe fruit production than plots with bottom nodes remaining, whereas the opposite was true for fruit titratable acidity. We see two ways to interpret these results. Titratable acidity often declines during ripening in *Vaccinium* fruits (Ismail & Kender, 1974), so high fruit acidity may reflect slowed ripening, and we have shown previously that high reproductive effort may slow ripening in lowbush blueberry (Bajcz & Drummond, in press). In that light, node success rate, ripe fruit production, and fruit ripening rate (with titratable acidity as a proxy) may all be lowbush blueberry traits limited by high reproductive effort because higher reproductive effort entails production of a greater number of more basal nodes, which may tend to have lower average quality.

On the other hand, organic acids can perform many positive functions inside fruit, including enhancing anthocyanin pigment color, preventing rotting, and, at least for humans, increasing taste complexity (Albert, Karp, Starast, Moor, & Paal, 2011; Ismail & Kender, 1974; Retamales & Hancock, 2012). More acidity could therefore equate with greater fruit attractiveness, palatability, and/or longevity and thus increase the likelihood the fruit is successfully dispersed. In that light, nodes produced at different positions could simply have alternative strengths—fruit produced near the apex may develop more quickly and effectively, whereas fruit produced more basally may be of higher average quality. Further experimentation will be needed to understand why this potential gradient of reproductive sink quality may exist in lowbush blueberry.

Our results are salient because while resource and sink limitations are often invoked to explain reproductive trade‐offs in plants (Brown & McNeil, 2006; Cao, Xie, Wu, & Yang, 2015; Guitián et al., 2001; Kudo & Molau, 1999; Lehtilä & Ehrlén, 2005; Trueman & Turnbull, 1994; Vallius, 2000; Wesselingh & Arnold, 2003), they are not the only plausible mechanisms. While we found some evidence that some of lowbush blueberry's reproductive trade‐offs may be linked to carbon and/or sink limitation, no form of limitation explained the full suite of trade‐offs we assessed here, and some trade‐offs (*e.g*., that between reproductive effort and fruit fresh:dry mass, discussed below) were not apparently caused by any of the forms of limitation we tested. This suggests we cannot assume, no matter how intuitive of explanations they may seem to be, that these forms of limitation *always* underlie plant reproductive trade‐offs without robust supporting evidence to back up these suppositions.

Perplexingly, fruit fresh:dry mass, which has consistently increased following flower removal in our studies (Bajcz & Drummond, in press), is not apparently caused by any form of limitation we tested here. Fruit dry mass is, by and large, composed of carbon in the forms of seeds, rind, and dissolved compounds (Stapanian, 1982). However, we found no evidence of carbon limitation for this trait. Furthermore, we have observed that mature seed number, fruit fresh mass (which largely reflects water mass), fruit sugar content, and fruit anthocyanin pigment content were all unaffected by flower removal (Bajcz & Drummond, in press). If the documented increases in fruit dry mass content in response to flower removal are not spurious but are not related to increased seed content or increased soluble carbon content in the form of sugars, anthocyanins, or organic acids, we are at a loss to propose a cause for this trade‐off.

### 4.2 Objective 2: best measures of reproductive effort for quantifying trade‐offs

For five of six traits studied, whether we had used our proportions of flower removal as our measure of reproductive effort or the number of nodes remaining per stem instead would not have affected our ability to observe a significant trade‐off. However, the decision of which measure to use did matter in terms of the apparent severity of those trade‐offs. For all traits, the slope of the trade‐off was more negative when the latter measure of reproductive effort was used. This was starkest for fruit dry:fresh mass; the standardized regression coefficients (β_std._) were approximately 40% apart for this trait between the competing models, suggesting that fruit dry mass content actually declined 40% faster with increasing reproductive effort than we would have otherwise predicted using the proportion of flower removal as our reproductive effort measure. Thus, while removal proportion may be a convenient and effective measure of reproductive effort, a more precise measure may produce more precise results in many cases.

### 4.3 Objective 3: reproductive trade‐off (non)linearity in lowbush blueberry

For five of six traits studied (all but proportion node success), an exponential function fit the trade‐off data at least as well as a linear function (Burnham & Anderson, 2002). For one of these traits, area per leaf, the exponential model fit the data better, indicating a trade‐off between this trait and reproductive effort that the linear model did not indicate. Thus, while linear models can be used to model trade‐offs, they may have limited ability to detect certain trade‐offs with very nonlinear shapes. It is true that nonlinear models like ours can be leveraged by extreme points. For this reason, it is crucial that every plausible level of trait expression, especially extreme levels, be captured and replicated when characterizing trade‐offs, if possible.

For most traits, the exponential model followed the linear one over much of the range of reproductive effort observed, and the fits were often similar between the competing models (Table 3). However, the departure points were enlightening in several ways. First, the departures were largest at the extremes of one or both traits (Figure 4a,b). Observing nonlinearity in many of these trade‐offs thus required including data from plots with extreme trait expression levels. When we chose lowbush blueberry clones for this study, we used perceivable phenotypic differences to include the most diverse set we could (Bell et al., 2010). Without doing so, we may not have captured the area‐per‐leaf trade‐off at all or the potential nonlinearity in trade‐offs like the one between ripe fruit per node and reproductive effort. Those wishing to characterize trade‐offs in a manner similar to ours may want to be similarly attentive to expression levels when they select their study individuals or populations.

Second, the departures may reflect ultimate limits to average lowbush blueberry trait expression levels. At some point, trait increases should be unsustainable, unprofitable, or cost‐ineffective (Valantin‐Morison et al., 2006). We found evidence of where some of these points may lie for lowbush blueberry; our exponential models indicated, for area per leaf (Figure 4a) and ripe fruit per node (Figure 4b), that these traits may reach their practical minimum or maximum within the range of reproductive effort we observed. Linear models, because they are not asymptotic, cannot reveal the location of these maxima or minima, so if one hopes to discern where these lie, use of a nonlinear approach will be necessary.

Lastly, the exponential models revealed that while two trade‐offs involving reproductive effort may be concave up (those between reproductive effort and ripe fruit per node and fruit dry:fresh mass), three others (those between reproductive effort and vegetative mass per stem, area per leaf, and fruit titratable acidity) may instead be concave down (Sletvold & Ågren, 2015). Putting these trade‐offs in context, a concave‐up form is thought to be evidence that a taxon is evolving to minimize the negative effects of (or to “opt out” of) a trade‐off (Sletvold & Ågren, 2015). Lowbush blueberry reproductive structures may ultimately depend on vegetative structures for carbon (Birkhold, Koch, & Darnell, 1992; Maust et al., 1999), so it is logical that lowbush blueberry would evolve to opt out of a vegetative‐effort‐versus‐reproductive‐effort trade‐off to the extent possible, even when these two functions necessarily compete for carbon initially (Sletvold & Ågren, 2015). Similarly, lowbush blueberry fruit may rot quickly because of their high sugar content (Glass, Percival, & Proctor, 2005) and may compete for limited dispersal opportunities (Bell et al., 2009), so it also logical that lowbush blueberry would opt out of a fruit‐longevity‐and‐attractiveness‐versus‐reproductive‐effort trade‐off as well. Still, our results indicate that even these traits begin to decline when a certain level of reproductive effort is reached—in other words, these trade‐offs cannot be entirely avoided.

Nor can lowbush blueberry opt out of every trade‐off; it must also “accept” some trade‐offs. Our analyses indicated that fruit ripening efficiency (as measured by ripe fruit production per node), reproductive node maintenance rate (as measured by proportion node success), and fruit dry mass content may be three traits that must decline relatively more steeply as reproductive effort increases. These results are consistent with current thoughts on resource prioritization and limitation (Brown & McNeil, 2006; Maust et al., 2000). If a plant can produce many fruit, selection may favor prioritizing the highest‐quality of these instead of equal investment across all of them (Brown & McNeil, 2006). It may also be physiologically impractical for all fruit to ripen quickly and simultaneously (Maust et al., 1999) or for each fruit to be equally and highly rewarding (Stapanian, 1982), necessitating steep trade‐offs between reproductive effort and traits like those identified above. If our results are supported by future work, we may have documented some of the selection pressures that underpin reproductive phenotypes in lowbush blueberry.

## 5 CONCLUSIONS AND FUTURE DIRECTIONS

As we look to the future, we encourage more continuous and precise measurement of reproductive effort in research of reproductive trade‐offs, although the best quantification of reproductive effort remains unclear. For example, is it best to use a measure of reproductive effort from the start of the growing season, as we did, or one from closer to harvest, when “realized reproductive effort,” so to speak, is better known (Sletvold & Ågren, 2015)? Additionally, is it best to quantify reproductive effort in terms of reproductive unit number, as we did, or in unit mass, as others (*e.g*., Thompson & Stewart, 1981) have proposed? Further, several studies (Aragón et al., 2009; Sletvold & Ågren, 2015; Bajcz & Drummond, in press) have noted that trade‐offs may only be significant under relatively stressful circumstances or may be evident only after repeated reductions in reproductive effort over multiple growing seasons (Ehrlén & van Groenendael, 2001). The 2‐year management cycle in lowbush blueberry (Bell et al., 2009) prevented us from subjecting the same clones to multiple years of removal, but we have already reported that the intensity of reproductive trade‐offs in lowbush blueberry varies between years, likely as a function of the stressfulness of prevailing abiotic conditions (Bajcz & Drummond, in press). In this light, the story we present here is by no means the whole story; characterizing reproductive trade‐offs fully includes examining when they do *not* occur and why as well (Sletvold & Ågren, 2015). Understanding when and why reproductive trade‐offs in plants are contextual should be a priority for future research.

In our study, sink limitation, not resource limitation, was the best explanation for why reproductive trade‐offs occur in lowbush blueberry of those we tested. Our definition of sink limitation, though perhaps convenient compared to the varied and decentralized meanings of its previous forms in the literature (Brown & McNeil, 2006; Emms, 1996; Guitián et al., 2001; Vallius, 2000; Wesselingh & Arnold, 2003), is still crude and hard to support unambiguously. In particular, the evidence needed to *fully* differentiate it from resource limitation, shy of demonstrating that *no* addition of *any* amount or type of *any* resource resolves performance differences between sinks, is still not clear. We welcome refinement of our definition to move the theory of plant reproductive ecology forward.

## CONFLICT OF INTEREST

None declared.

## AUTHOR'S CONTRIBUTIONS

AWB and FAD designed the experiment; AWB carried out the fieldwork and laboratory analyses and wrote the manuscript; FAD provided editorial guidance and financially supported the research. Both authors revised the manuscript and gave final approval for publication.
